# Purposeful selection of variables in logistic regression

**DOI:** 10.1186/1751-0473-3-17

**Published:** 2008-12-16

**Authors:** Zoran Bursac, C Heath Gauss, David Keith Williams, David W Hosmer

**Affiliations:** 1 Biostatistics, University of Arkansas for Medical Sciences, Little Rock, AR 72205, USA; 2 Biostatistics, University of Massachusetts, Amherst, MA 01003, USA

## Abstract

**Background:**

The main problem in many model-building situations is to choose from a large set of covariates those that should be included in the "best" model. A decision to keep a variable in the model might be based on the clinical or statistical significance. There are several variable selection algorithms in existence. Those methods are mechanical and as such carry some limitations. Hosmer and Lemeshow describe a purposeful selection of covariates within which an analyst makes a variable selection decision at each step of the modeling process.

**Methods:**

In this paper we introduce an algorithm which automates that process. We conduct a simulation study to compare the performance of this algorithm with three well documented variable selection procedures in SAS PROC LOGISTIC: FORWARD, BACKWARD, and STEPWISE.

**Results:**

We show that the advantage of this approach is when the analyst is interested in risk factor modeling and not just prediction. In addition to significant covariates, this variable selection procedure has the capability of retaining important confounding variables, resulting potentially in a slightly richer model. Application of the macro is further illustrated with the Hosmer and Lemeshow Worchester Heart Attack Study (WHAS) data.

**Conclusion:**

If an analyst is in need of an algorithm that will help guide the retention of significant covariates as well as confounding ones they should consider this macro as an alternative tool.

## Background

The criteria for inclusion of a variable in the model vary between problems and disciplines. The common approach to statistical model building is minimization of variables until the most parsimonious model that describes the data is found which also results in numerical stability and generalizability of the results. Some methodologists suggest inclusion of all clinical and other relevant variables in the model regardless of their significance in order to control for confounding. This approach, however, can lead to numerically unstable estimates and large standard errors. This paper is based on the purposeful selection of variables in regression methods (with specific focus on logistic regression in this paper) as proposed by Hosmer and Lemeshow [1,2].

It is important to mention that with the rapid computing and information evolution there has been a growth in the field of feature selection methods and algorithms. Some examples include hill-climbing, greedy algorithms, recursive feature elimination, univariate association filtering, and backward/forward wrapping, to name a few. These methods have been used in bioinformatics, clinical diagnostics, and some are universal to multiple applications. Hill-climbing and greedy algorithms are mathematical optimization techniques used in artificial intelligence, which work well on certain problems, but they fail to produce optimal solutions for many others [3-6]. Filtering, wrapping, and recursive feature elimination methods have been used in areas like text processing or gene expression array analysis. While these are powerful selection methods that have improved the performance of predictors, they are often computationally intensive. They are used on large data sets often with thousands of variables, introducing the problem of dimensionality and like some other multivariate methods have potential to overfit the data [7].

Several variable selection methods are available in commercial software packages. Commonly used methods, which are the ones of focus in this paper, are forward selection, backward elimination, and stepwise selection.

In forward selection, the score chi-square statistic is computed for each effect not in the model and examines the largest of these statistics. If it is significant at some entry level, the corresponding effect is added to the model. Once an effect is entered in the model, it is never removed from the model. The process is repeated until none of the remaining effects meet the specified level for entry.

In backward elimination, the results of the Wald test for individual parameters are examined. The least significant effect that does not meet the level for staying in the model is removed. Once an effect is removed from the model, it remains excluded. The process is repeated until no other effect in the model meets the specified level for removal.

The stepwise selection is similar to the forward selection except that effects already in the model do not necessarily remain. Effects are entered into and removed from the model in such a way that each forward selection step may be followed by one or more backward elimination steps. The stepwise selection process terminates if no further effect can be added to the model or if the effect just entered into the model is the only effect removed in the subsequent backward elimination

The purposeful selection algorithm (PS) follows a slightly different logic as proposed by Hosmer and Lemeshow [1,2]. This variable selection method has not been studied or compared in a systematic way to other statistical selection methods, with the exception of a few numerical examples.

An important part of this study was the development and validation of a SAS macro that automates the purposeful selection process. Details on the macro and the link to macro itself are provided in the appendix. Since the macro was written in SAS, we compare its performance with SAS PROC LOGISTIC variable selection procedures, namely FORWARD (FS), BACKWARD (BS), and STEPWISE (SS) [8].

The objectives of this paper are 1) to evaluate the purposeful selection algorithm systematically in a simulation study by comparing it to the above mentioned variable selection procedures, and 2) to show the application of it on the motivating data set.

### Purposeful selection of covariates

The purposeful selection process begins by a univariate analysis of each variable. Any variable having a significant univariate test at some arbitrary level is selected as a candidate for the multivariate analysis. We base this on the Wald test from logistic regression and p-value cut-off point of 0.25. More traditional levels such as 0.05 can fail in identifying variables known to be important [9,10]. In the iterative process of variable selection, covariates are removed from the model if they are non-significant and not a confounder. Significance is evaluated at the 0.1 alpha level and confounding as a change in any remaining parameter estimate greater than, say, 15% or 20% as compared to the full model. A change in a parameter estimate above the specified level indicates that the excluded variable was important in the sense of providing a needed adjustment for one or more of the variables remaining in the model. At the end of this iterative process of deleting, refitting, and verifying, the model contains significant covariates and confounders. At this point any variable not selected for the original multivariate model is added back one at a time, with significant covariates and confounders retained earlier. This step can be helpful in identifying variables that, by themselves, are not significantly related to the outcome but make an important contribution in the presence of other variables. Any that are significant at the 0.1 or 0.15 level are put in the model, and the model is iteratively reduced as before but only for the variables that were additionally added. At the end of this final step, the analyst is left with the preliminary main effects model. For more details on the purposeful selection process, refer to Hosmer and Lemeshow [1,2].

### Simulations

We conducted two simulation studies to evaluate the performance of the purposeful selection algorithm. In the first simulation we started with the assumption that we have 6 equally important covariates *(X*_1_, ..., *X*_6 _such that *X*_*j*_~*U*(-6, 6) for *j *= 1, ..., 6), three of which were significant and three that were not. We set *β*_0 _= -0.6, *β*_1 _= *β*_2 _= *β*_3 _= 0.122, and *β*_4 _= *β*_5 _= *β*_6 _= 0. Therefore, the true logit we sampled from was

*logit *= -0.6 + 0.122*X*_1 _+ 0.122*X*_2 _+ 0.122*X*_3 _+ 0*X*_4 _+ 0*X*_5 _+ 0*X*_6_.

We conducted 1000 simulation runs for each of the 6 conditions in which we varied the sample size (n = 60, 120, 240, 360, 480, and 600). The summary measure of the algorithm performance was the percent of times each variable selection procedure retained only *X*_1_, *X*_2_, and *X*_3 _in the final model. (For PS selection, confounding was set to 20% and non-candidate inclusion to 0.1, even though confounding was not simulated in this portion of the study.)

Table 1 shows the percent of times that the correct model was obtained for four selection procedures under various sample sizes. Correct retention increases with sample size, and it is almost identical for PS, SS, and BS. FS selection does not perform as well as the other three with the exception of lower sample size levels.

**Table 1:** Simulation results.

n	Purposeful	Stepwise	Backward	Forward
60	5.1	4.5	4.9	9
120	24.2	22.4	22.8	24.2
240	52.6	52.6	52.5	36
360	69.8	69.8	69.8	42.5
480	71.1	71.2	71.1	44.2
600	70.5	70.6	70.5	40.4

Retention of the correct model for purposeful, stepwise, backward, and forward selection methods, with no confounding present.

In the second simulation, we started with the same assumption, that the 6 covariates were equally important, two of which were significant, one that was a confounder, and three that were not significant. We assumed that *X*_1 _= Bernoulli (0.5), the confounder *X*_2_~*U*(-6, 3) if *X*_1 _= 1 and *X*_2_~*U*(-3, 6) if *X*_1 _= 0, and *X*_3 _- *X*_6_~*U*(-6, 6). We created the confounder *X*_2 _by making the distribution of that variable dependent on *X*_1_. We set *β*_0 _= -0.6, *β*_1 _= 1.2, *β*_2 _= 0.1, *β*_3 _= 0.122, and *β*_4 _= *β*_5 _= *β*_6 _= 0. Therefore, the true logit we sampled from was

*logit *= -0.6 + 1.2*X*_1 _+ 0.1*X*_2 _+ 0.122*X*_3 _+ 0*X*_4 _+ 0*X*_5 _+ 0*X*_6_.

We conducted 1000 simulation runs for each of the 24 conditions in which we varied the sample size (n = 60, 120, 240, 360, 480, and 600), confounding (15% and 20%), and non-candidate inclusion (0.1 and 0.15). Similarly, the summary measure of the algorithm performance was the percent of times each variable selection procedure retained only *X*_1_, *X*_2_, and X_3 _in the final model.

Table 2 shows the percent of times that the correct model was obtained for four selection procedures under 24 simulated conditions.

**Table 2:** Simulation results

Confounding	Non-candidate Inclusion	n	Purposeful	Stepwise	Backward	Forward
20	0.1	60	5	3.6	6.3	9.1
		120	17.3	15.6	18.2	18.8
		240	39.7	39.6	40.1	30.3
		360	55.2	54.4	54.4	36.6
		480	64.3	64.3	64.3	37.5
		600	65.8	65.7	65.7	41.3
						
20	0.15	60	9.2	4.6	6.4	8.1
		120	18.7	14.8	17.2	18.5
		240	43.1	37.1	38.2	30.5
		360	56.5	53.7	53.9	37
		480	63.6	62.6	62	43
		600	70.3	69	68.7	41
						
15	0.1	60	6.6	4.1	6.1	9.6
		120	17.8	15.6	18.6	19.2
		240	39.7	36.6	37.6	29.8
		360	53.3	52.2	52.6	38.3
		480	62.4	62.1	62.1	40.1
		600	68.5	67.9	68	40.2
						
15	0.15	60	9.7	4.4	6.7	9
		120	21.9	16.8	21.3	19.6
		240	46.6	40.2	41.4	32.3
		360	57.7	52.5	52.5	35.3
		480	64	63.1	63.1	39.3
		600	70.4	69.6	69.6	41.4

Retention of the correct model for purposeful, stepwise, backward, and forward selection methods, under 24 simulated conditions that vary confounding, non-candidate inclusion, and sample size levels.

Again, the proportion of correctly retained models increases with sample size for all selection methods. At the lower sample size levels no procedure performs very well. FS does the best with the exceptions when the non-candidate inclusion is set to 0.15, where PS performs better. With the larger samples like 480 and 600, PS, SS, and BS converge toward a close proportion of correct model retention while FS does notably worse. With confounding present, PS retains a larger proportion of correct models for all six sample sizes when confounding is set to either 15% or 20% and non-candidate inclusion to 0.15 as compared to the other three methods. Under the other scenarios, PS retains a slightly larger proportion of correct models than the other variable selection procedures, mainly for samples in the range 240–360.

In addition to the mentioned simulation conditions, we tampered with the coefficient of the confounding variable *X*_2_, by making it more significant at 0.13, and less significant at 0.07. We show the results for both scenarios with confounding set to 15% and non-candidate inclusion at 0.15.

When *β*_2 _= 0.13, Table 3 shows that PS, BS, and as sample size gets larger, SS perform comparably, retaining a similar proportion of correct models. This is primarily due to the fact that *X*_2 _becomes significant in a larger proportion of simulations and is retained by those procedures because of its significance and not confounding effect. FS again mostly does worse than the three previously mentioned selection procedures.

**Table 3:** Simulation results.

*β*_2_	n	Purposeful	Stepwise	Backward	Forward
0.13	60	9.7	6.3	10.3	10.8
	120	25.8	19.8	24.9	23
	240	55.5	52	54.9	37.4
	360	66.4	65.5	65.8	38.7
	480	72.5	72.7	72.8	41.1
	600	71.4	72.9	72.9	42.9
					
0.07	60	7.5	3.1	4.4	6.7
	120	18.6	11.3	12.2	15.8
	240	32.2	22.5	22.9	21.4
	360	41.5	35.5	35.5	26.9
	480	47.9	44.5	44.5	34.6
	600	52	50.5	50.5	35.5

Retention of the correct model for purposeful, stepwise, backward, and forward selection methods, for two values of *β*_2 _while specifying confounding at 15% and non-candidate inclusion at 0.15.

When *β*_2 _= 0.07, Table 3 shows that PS performs better across all sample sizes than other variable selection procedures; however, the proportion of correctly retained models is lower for all procedures. This is a result of the fact that *X*_2 _becomes non-significant in more simulations and is not retained. Table 3 also shows how *X*_2 _is picked up by PS due to its confounding effect which is still present.

## Application

A subset of observations (N = 307) and variables from the Worchester Heart Attack Study (WHAS) data set [1,11,12] were used to compare the results of variable selections between the purposeful selection method and each of the three methods available in SAS PROC LOGISTIC as described above. Variable inclusion and exclusion criteria for existing selection procedures in SAS PROC LOGISTIC were set to comparable levels with the purposeful selection parameters. Specifically, the variable entry criterion was set to 0.25 and the variable retention criterion to 0.1 to minimize the discrepancies as a result of non-comparable parameters.

The main outcome of interest was vital status at the last follow-up, dead (FSTAT = 1) versus alive (FSTAT = 0). The eleven covariates listed in Table 4 were treated as equally important. The macro calls used to invoke purposeful selection of variables from the WHAS data set under different confounding and non-candidate inclusion settings are given in the appendix.

**Table 4:** WHAS data set variables.

FSTAT	Status as of last follow-up (0 = Alive, 1 = Dead)
AGE	Age at hospital admission (Years)
SEX	Gender (0 = Male, 1 = Female)
HR	Initial heart rate (Beats per minute)
BMI	Body mass index (kg/m^2^)
CVD	History of cardiovascular disease (0 = No, 1 = Yes)
AFB	Atrial fibrillation (0 = No, 1 = Yes)
SHO	Cardiogenic shock (0 = No, 1 = Yes)
CHF	Congestive heart complications (0 = No, 1 = Yes)
AV3	Complete heart block (0 = No, 1 = Yes)
MIORD	MI order (0 = First, 1 = Recurrent)
MITYPE	MI type (0 = non - Q-wave, 1 = Q-wave)

Table 5 shows the results of variable retention from our macro and PROC LOGISTIC selection procedures. The univariate analysis identified 9 covariates initially as potential candidates for the multivariate model at the 0.25 alpha level based on the Wald chi-square statistic. Those included AGE, SEX, HR, BMI, CVD, AFB, SHO, CHF, and MIORD. During the iterative multivariate fitting, four of them (SEX, CVD, AFB, and CHF) were eliminated one at a time because they were not significant in the multivariate model at the alpha level of 0.1, and when taken out, did not change any remaining parameter estimates by more than 20%. The variable BMI was also not significant at the 0.1 alpha level but changed the parameter estimate for the MIORD covariate by more than 20% when taken out; therefore, it remained in the model as a confounder. The maximum p-value of the remaining variables AGE, SHO, HR, and MIORD was less than 0.1, at which point the variables originally set aside were reconsidered.

**Table 5:** WHAS data set variables retained in the final models for purposeful selection method under two different settings.

*Purposeful Selection* *(20%, 0.1)*	*p-value*	*Purposeful Selection* *(15%, 0.15)*	*p-value*	*Forward, Backward, Stepwise*	*p-value*
AGE	<0.0001	AGE	<0.0001	AGE	<0.0001
SHO	0.0018	SHO	0.0029	SHO	0.0039
HR	0.0025	HR	0.0019	HR	0.0011
MITYPE	0.091	MITYPE	0.0586	MITYPE	0.0149
MIORD	0.1087	AV3	0.0760	AV3	0.0672
BMI	0.2035	MIORD	0.1285		
		BMI	0.2107		

SAS PROC LOGISTIC forward, backward, and stepwise selection methods.

Out of the remaining two variables set aside initially because they were not significant at the 0.25 level (AV3 and MITYPE), MITYPE made it back in the model when tested (one at a time) with the five retained covariates because it was significant at the 0.1 alpha level. The addition of MITYPE confounded the relationship between MIORD and FSTAT, hence the change in the MIORD p-value from 0.0324 to 0.1087.

All three selection procedures available in SAS PROC LOGISTIC resulted in the same model (Table 5). While the resulting model contains only significant covariates, it did not retain the confounder BMI or the variable MIORD which were retained by the purposeful selection method. On the other hand, the variable AV3 was retained.

Changing the value of confounding to 15% and non-candidate inclusion to 0.15 resulted in the addition of the variable AV3, which was a non-candidate originally but made it in the model at the higher non-candidate inclusion level since its significance was 0.1173. This particular specification resulted in the exact variables that were retained by available selection procedures in SAS PROC LOGISTIC with the addition of one confounding variable (BMI) and another potentially important covariate (MIORD).

## Discussion

The human modeling process still remains an effective one. We can attempt to control for as many situations as possible through automated computer algorithms, but that is still not an adequate replacement for a skilled analyst making decisions at each step of the modeling process.

The advantage of the purposeful selection method comes when the analyst is interested in risk factor modeling and not just mere prediction. The algorithm is written in such a way that, in addition to significant covariates, it retains important confounding variables, resulting in a possibly slightly richer model.

The simulation study demonstrates that the purposeful selection algorithm identifies and retains confounders correctly at a larger rate than other selection procedures, particularly in instances where the significance level of a confounder is between 0.1 and 0.15, when the other algorithms would not retain it.

We realize that many studies have samples much larger than 600. We tested larger sample sizes, 1000 for instance, and the simulation results suggest that all selection methods except FS converge toward the same proportion of correctly retained models. As the sample gets larger, the variability of even borderline significant confounders gets smaller, and they get retained as significant variables, hence diminishing the retention differences between the selection methods. It is evident from the simulation results that PS works well for the samples in the range of 240–600, a common number of participants in epidemiologic and behavioral research studies.

### Limitations

There are a few limitations to this algorithm. First, variables not selected initially for the multivariate model are tested later on with the selected set of covariates one at a time. This is a possible limitation because any of these variables that would be significant when put in the model jointly will be missed. However, being significant jointly may indicate multicollinearity, in which case the analyst may choose to use only one of those as a proxy or not at all. Also if there is some multicollinearity between significant variables they would likely be retained by all selection procedures as a result of their significant effect. Second, if two non-significant covariates confound each other, they are going to be retained as confounders since all covariates are assumed to be equally important. In a situation where that happens, the analyst should probably consider retaining the two covariates if they are significant at the 0.25 level, indicating some reasonable association with the outcome. Otherwise, the analyst should probably exclude both from the model as meaningless confounders. Additionally, if there is some multicollinearity between non-significant variables, they would likely be retained by PS as a result of confounding effect on each other, and missed by other three selection procedures as a result of their non-significant effect. Third, this algorithm was not designed to force all dummy variables in the model (for instance, one that has three nominal levels which corresponds to two dummy variables that need to be considered as a unit in model inclusion), if one is significant. Other selection procedures have this limitation as well, unless you force dummy variables in the model. However, it is not possible to know a priori whether one of the dummy variables will be significant. If one of the dummy variables is retained as significant, the analyst can manually insert the rest of them in the model. Finally, multi-class problems were not explored in this paper; therefore, the results do not support the robustness of PS over a range of model selection applications and problems.

## Conclusion

If an analyst is in need of an algorithm that will help guide the retention of significant covariates as well as confounding ones, this macro will provide that. In order to improve the chances of retaining meaningful confounders, we recommend setting the confounding level to 15% and the non-candidate inclusion level to 0.15. Analysts should use this macro as a tool that helps with decisions about the final model, not as a definite answer. One should always carefully examine the model provided by this macro and determine why the covariates were retained before proceeding.

## Appendix

### *%PurposefulSelection *SAS Macro Description

**Figure 1:**
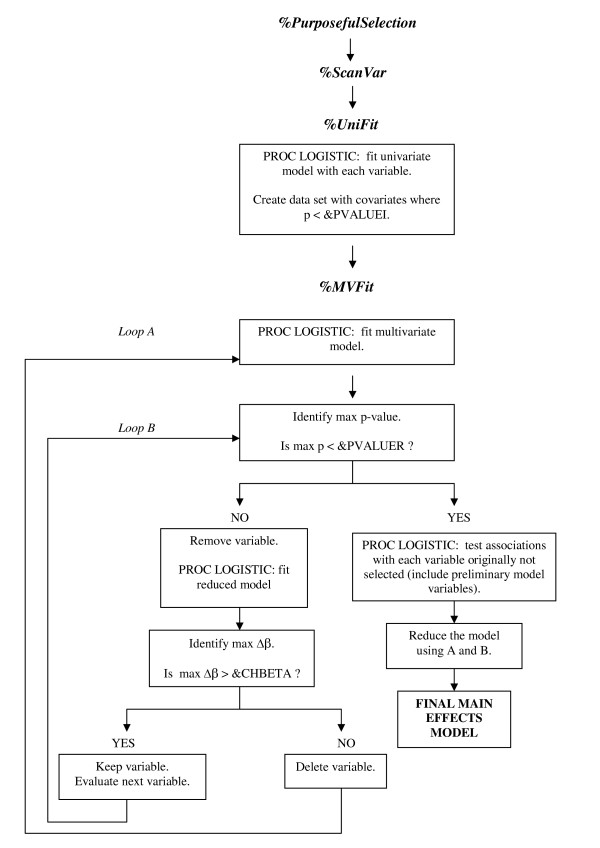
***%PurposefulSelection *****macro flow chart.**

The main *%PurposefulSelection *(PS) macro consists of three calls to sub-macros, *%ScanVar*, *%UniFit*, and *%MVFit*. The *%ScanVar *sub-macro scans the submitted covariates and prepares them for the univariate analysis. The *%UniFit *sub-macro fits all univariate models and creates a data set with the candidate variables for the multivariate analysis. The *%MVFit *sub-macro iteratively fits multivariate models while evaluating the significance and confounding effect of each candidate variable as well as those that were not originally selected. A flowchart of the macro is presented in Figure 1.

The user must define several macro variables as shown in Table 6. The macro variable *DATASET *corresponds to the data set to be analyzed. Macro variable *OUTCOME *is the main outcome of interest and should be a binary variable (also known as the dependent variable). The macro uses the *DESCENDING *option by default to model the probability of *OUTCOME *= 1. The macro variable *COVARIATES *represents a set of predictor variables which can all be continuous, binary, or a mix of the two. In the case of a polytomous covariate, dummy variables must be created before invoking the macro and specified as separate variables. All covariates specified here are assumed to be of equal importance. The macro variable *PVALUEI *defines the alpha level for the univariate model at which a covariate will be considered as a candidate for the multivariable analysis. The macro variable *PVALUER *defines the retention criterion for the multivariate model at which a variable will remain in the model. The macro variable *CHBETA *represents the percent change in a parameter estimate (beta) above which a covariate that is removed from the model as non-significant will be considered a confounder and placed back in the model. Even though we recommend inclusion and retention criteria to be set at 0.25 and 0.1, respectively, and confounding at 15% change, these parameters can be directly controlled by the analyst, since they are coded as macro variables. Finally, the macro variable *PVALUENC *defines the inclusion criterion for any non-candidate variables, allowing them to make it back into the model. We recommend this value be set at 0.15 for reasons discussed in the simulation study and application sections. [See additional file 1: Purposeful Selection Macro v Beta1_1.txt]

**Table 6:** *%PurposefulSelection *macro variables.

DATASET	Input data set
OUTCOME	Main outcome (Y)
COVARIATES	All covariates (X_1_...X_*j*_)
PVALUEI	Inclusion criteria for multivariate model
PVALUER	Retention criteria for multivariate model
CHBETA	% change in parameter estimate indicating confounding
PVALUENC	Inclusion criteria for non-candidate

### *%PurposefulSelection *SAS Macro Code

Link to SAS macro code: 

Two macro calls used to analyze WHAS data set described in the application section:

%***PurposefulSelection ***(whas, fstat, age sex hr bmi cvd afb sho chf av3 miord mitype, **0.25**, **0.1**, **20, 0.1**);

%***PurposefulSelection ***(whas, fstat, age sex hr bmi cvd afb sho chf av3 miord mitype, **0.25**, **0.1**, **15, 0.15**);

## Competing interests

The authors declare that they have no competing interests.

## Authors' contributions

ZB coded and tested the macro and wrote the manuscript. CHG coded and tested the macro and worked on revisions of the paper. DKW helped with conceptual design of the idea for the algorithm and worked on the revisions of the manuscript. DWH conceptualized the idea for the macro and worked on the revisions of the paper.

## Supplementary Material

Additional file 1 **Purposeful Selection Macro v Beta1_1.txt** Click here for file
